# Small Bowel Adenocarcinoma: 10-Year Experience in a Cancer Center—The Ottawa Hospital (TOH)

**DOI:** 10.3390/curroncol29100585

**Published:** 2022-10-05

**Authors:** Abdulhameed Alfagih, Mohammad Alrehaili, Timothy Asmis

**Affiliations:** 1 Division of Medical Oncology, Department of Medicine, The Ottawa Hospital, The University of Ottawa, Ottawa, ON K1H 8L6, Canada; 2 Medical Oncology Department, Comprehensive Cancer Center, King Fahad Medical City, Riyadh 11525, Saudi Arabia

**Keywords:** small bowel neoplasm, adenocarcinoma, survival, chemotherapy

## Abstract

(1) *Background:* Small bowel adenocarcinoma (SBA) is one of the predominant primary small bowel cancers that has a dismal outcome. We aim to report 10 years of experience in SBA management at a regional cancer centre in Canada.; (2) *Methods:* We retrospectively analysed clinical and pathological data of patients diagnosed with an SBA between 2011 and 2021 at the Ottawa Hospital (TOH), Ottawa, Canada. We describe the clinicopathological features and outcomes, including survival. Potential prognostic factors were analysed using the Cox proportional hazard model for multivariate analysis.; (3) *Results:* We identified 115 patients with SBA. The duodenum was the most common SBA location representing 61% (70) of the total patients, followed by the jejunum (17%) and ileum (10%). Around 24% (27) of cases presented with bowel obstructions. The majority of patients (56%, 64) had stage IV disease on presentation. Seven patients had MSI-high tumours, while 24% (27) were MS-stable. In terms of management, 48 patients underwent curative surgical resection, 17 of whom received adjuvant chemotherapy. On the other hand, 57 patients (49.5%) with the advanced disease received palliative systemic therapy, and 18 patients (16%) had supportive care only. Over a median follow-up of 21.5 months (range 0–122), the median overall survival was 94, 61, and 34 months for stages II, III, and IV, respectively (*p* < 0.05). The median recurrence-free survival was 93 and 23 months for stages II and III, respectively. However, there was no statistically significant difference between TNM stages in RFS, *p* = 0.069. Multivariate Cox regression analysis showed only poor performance status at diagnosis as a predictor for shorter overall survival (*p* < 0.05). The univariate analysis didn’t show any significant correlation between RFS and covariants.; (4) *Conclusions:* SBA remains one of the most aggressive tumours with a dismal prognosis even after surgical resection. The optimal chemotherapy regimen has not been established. Further studies are needed to explore the role of adjuvant chemotherapy for stages I-III SBA.

## 1. Introduction

Primary Small bowel cancer (SBC) is an uncommon malignancy representing 3–4% of all gastrointestinal tract (GI) cancers. Nevertheless, SBC incidence was noted to be increasing over the last two decades, with an incidence of 2.3 per 100,000 and a median age of 66 years at diagnosis [1]. Small bowel adenocarcinoma (SBA) accounts for a 30–40% incidence of SBC. It carries a poor prognosis compared to other SBC histologic subtypes, with overall survival of around 10% for stage IV and about 63–32% for stages I–III [2]. Around 50% of SBCs arise in the duodenum, followed by the jejunum (30%) and ileum (15%) [3]. SBA is often associated with advanced age, inflammatory bowel disease (IBD), and coeliac disease. However, it can also be caused by specific inheritable syndromes such as familial adenomatosis polyposis (FAP) syndrome, Lynch syndrome, and MUTYH-associated polyposis (MAP) [4,5,6,7].

The pathogenesis of SBA is not well understood; however, there is evidence that suggests some similarities between SBA and colorectal cancer (CRC). Mutations in p53, β -CATENIN, APC, BRAF, and MMR genes have been implicated in SBA development similarly to CRC [8,9,10]. Moreover, the KRAS mutation rate in SBA is comparable to that observed in CRC (40–60%), while BRAF mutations are infrequent in SBA [11]. 

The prognosis of patients with SBA is often poor. Several factors associated with a poor prognosis in SBA were reported, such as male gender, T4 tumours, lymph node (LN) involvement, poor differentiation, metastatic disease, and lymphovascular invasion [12]. In contrast, mismatch repair deficiency is identified as a significant predictor of favourable cancer-specific survival [13].

Nearly two-thirds of SBC patients (predominantly those with adenocarcinoma histology) present acutely with either perforation or bowel obstruction. Unfortunately, 50% of SBC patients have the advanced disease on presentation owing to vague non-specific symptoms resulting in a delay of diagnosis with a mean duration of symptoms before diagnosis around 10 months [2,14].

SBA is a challenging disease to treat, and its management is based on the site and stage of disease at presentation, patient comorbidities, performance status, and available expertise. Surgical resection is the mainstay of management for resectable disease. Nevertheless, approximately 64% of SBC patients could undergo a complete resection. SBA has (62%) potential for curative resection. Complete resection offers the longest survival and is considered a significant prognostic predictor of overall survival. The recurrence of SBA is common, and the outcome after recurrence is dismal [15,16,17,18].

There is a dearth of data regarding the experience with SBA, and the purpose of this study was to report 10-year experiences with primary SBA in The Ottawa Hospital (TOH).

## 2. Materials and Methods

### 2.1. Study Design and Setting

This is an observational retrospective cohort study. We identified SBA cases by searching hospital databases using ICD 10 codes. We examined records of all SBA cases referred to or diagnosed in TOH between 01 January 2011, and 31 December 2021.

### 2.2. Study Inclusion Criteria

All adult patients (age >18) with biopsy-proven diagnoses of SBA were included in the study.

### 2.3. Study Exclusion Criteria

Cases without tissue diagnosis were excluded.

### 2.4. Clinical Data Extraction 

Details of the tumour site, stage (based on American Joint Committee on Cancer (AJCC) TNM Staging Classification for Small Intestine Adenocarcinoma 8th ed), clinical, pathological, management and outcomes data were recorded. Data were collected from the electronic medical records (Epic) on the access database form.

### 2.5. Statistical Analysis

Results were analysed using MS Excel and SPSS 25.0 software used for data analysis. Descriptive statistics were used to summarise data and synthesise and report patients’ demographic and clinicopathological data. Qualitative variables were analysed by the χ2 test and Fisher’s exact test. Survival data (RFS, PFS, OS) were analysed using Kaplan–Meier methods and compared using the log-rank test. OS was calculated from the date of tissue diagnosis to the date of death or last follow-up. Recurrence-free survival (RFS) was calculated from the date of surgical intervention to the date of recurrence or death or last follow-up. Potential prognostic factors were analysed using the Cox proportional hazard model for multivariate analysis. The 2-tailed *p*-values were reported and were considered to be statistically significant when *p* < 0.05.

## 3. Results

### 3.1. Patient Characteristics

The patient’s characteristics are shown in Table 1. In total, 115 patients were identified with an average age of 67 (range 40–89) years, with males representing 58% (*n* = 67). An average of 12 cases were diagnosed per year (range 6–17).

### 3.2. SBA Sites 

The most common primary site was the duodenum which was involved in 61% (*n* = 70) of patients. The jejunum and ileum represented 17% and 10%, respectively. The duodenum, compared to other SBA sites, had a higher incidence of non-metastatic disease at presentation (*p* = 0.001) and less IBD (*p* = 0.033). There was no significant association between SBA sites (duodenum vs. Others) and gender, recurrence rate, PNI, LVI, and secondary malignancy.

### 3.3. Clinical Presentation

Around 29% had symptoms for more than 6 months, while 14% (*n* = 16) presented with acute symptoms for less than 14 days. Nearly 48% (*n* = 55) of patients had abdominal pain as the main presenting symptom, while anaemia was the chief complaint in 25% (*n* = 29) of cases. Small bowel obstruction or gastric outlet obstruction were the main presentation in 24% (*n* = 27) and 14 % (*n* = 16) of patients, respectively. Approximately 28% (*n* = 25) of patients had a positive family history of GI cancer. However, only six patients had a diagnosis of the familial syndrome. IBD was documented in six patients. In around 45% (*n* = 52) of cases, the initial diagnosis was made using CT, while 23% (*n* = 26) were diagnosed initially by endoscopy. Fifteen patients presented with recurrent small bowel obstruction, representing 13% of all cases.

### 3.4. Pathology Data

The pathology and staging date are shown in Table 2. The majority of patients (56%, *n* = 64) had stage IV disease on presentation, while only eight patients had stage I at the time of diagnosis. Among the patients with stage IV disease, the most common sites of metastasis were peritoneum, liver, lung and lymph nodes representing 31%, 26%, 18% and 12%, respectively.

### 3.5. MSI, RAS, BRAF 

The majority of the patients had unknown RAS (82%), BRAF (82%) or MSI (70%) status. Nine percent of patients (*n* = 10) had RAS mutation, and 3% (three) had BRAF mutation. Seven patients had MSI – high tumours, while 24% (*n* = 27) had MS stable.

### 3.6. Management and Outcomes

**Figure 1:**
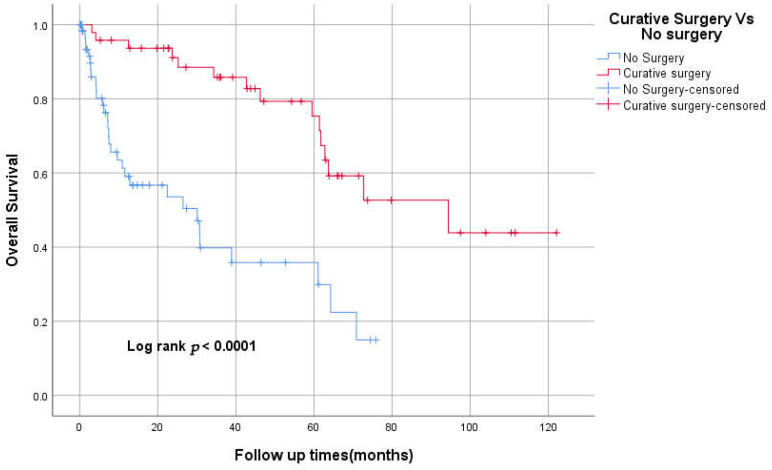
Kaplan–Meier curve of Overall survival for curative surgery vs. no surgery.

Forty-eight patients (42%) underwent curative surgical resection. The median OS for the curative surgery group compared to the no-surgery group was 94.4 Vs 30.1 months, respectively (Figure 1). Nine patients of the curative surgery group had mastectomies, four patients of whom received conversion-type chemotherapy. Among patients with stages II and III, 17 patients only received adjuvant chemotherapy, including FOLFOX, Capecitabine and CAPOX for 10, three and two patients, respectively. Adjuvant concurrent chemoradiation followed by 5-FU and 5-FU only were given for the remaining two patients. Eight patients had recurrence after adjuvant chemotherapy.

Among the patients with stage IV disease, 57 patients (49.5%) received palliative systemic therapy, while 16% (*n* = 18) had supportive care only. Only 10 patients received three lines or more of systemic therapy.

### 3.7. Secondary Malignancy

Twenty-eight (24%) patients had a diagnosis of additional primary cancer prior to or after SBA diagnosis. Among them, 10 patients (36%) had colon cancer. 

### 3.8. Survival

The Median follow-up was 21.5 months (range 0–122). In the entire study cohort, death was documented in 46 patients (40%). There were 33 (29%) patients who likely died (e.g., transferred to hospice or home palliation); however, death was not confirmed in the medical records. These patients were reported as alive as of the last known follow-up. 

**Figure 2:**
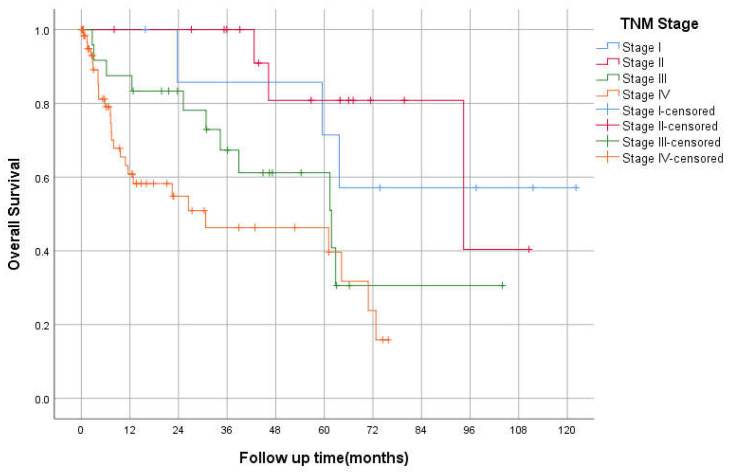
Kaplan–Meier curve of Overall Survival based on TNM Staging.

**Figure 3:**
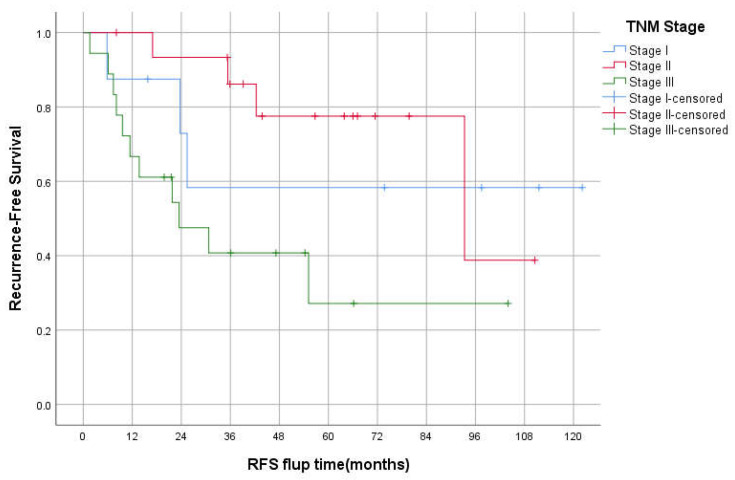
Kaplan–Meier curve of Recurrence-free survival for stages I-III.

**Figure 4:**
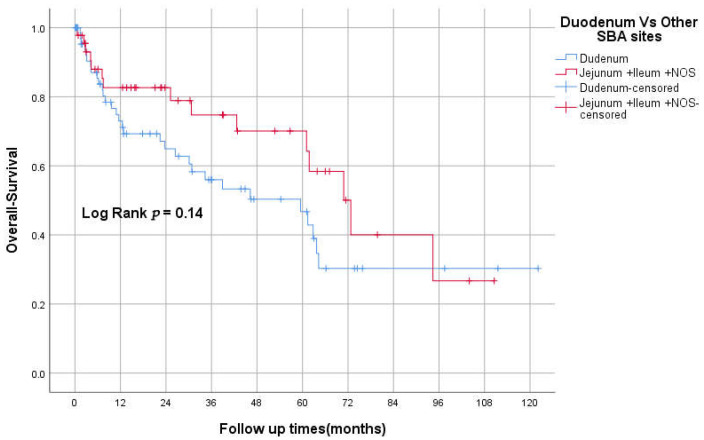
Kaplan–Meier curve of OS for duodenum vs. other SBA sites.

The median OS was 94 (95% CI 26–162), 61 (95% CI 31–92), and 34 (95% CI 0–74) months for stages II, III, and IV, respectively (*p* < 0.05; Figure 2). The 3-year OS was 87%, 82%, 60%, 50% for stages I–IV respectively. The median RFS was 93 and 23 months for stages II and III, respectively. However, there was no statistically significant difference between TNM stages in RFS, *p* = 0.069 (Figure 3). The 3-years RFS was 59%, 78% and 42% for stages I–III, respectively. The duodenum, compared to other SBA sites, had no statistically significant difference in OS (log-rank, *p* = 0.14; Figure 4).

### 3.9. 1st Line FOLFOX vs. FOLFIRI 

**Figure 5:**
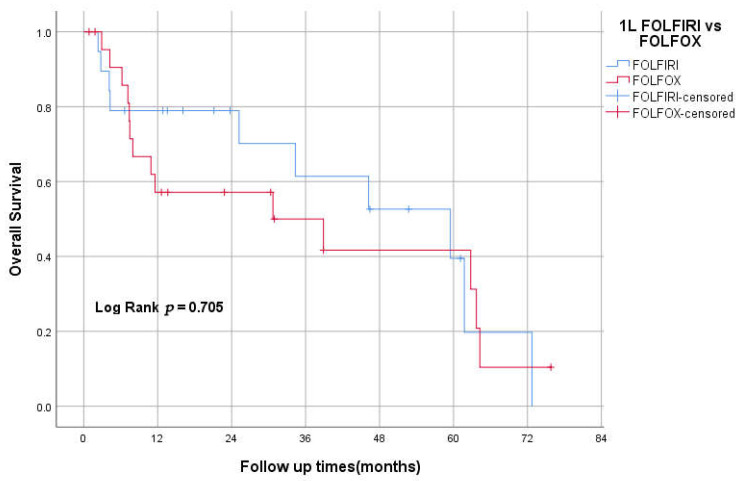
Kaplan–Meier curve of OS for first-line FOLFOX vs. FOLFIRI.

Nineteen patients received FOLFIRI as an initial treatment, while 23 received FOLFOX. The median OS for the FOLFIRI group was 59 months (95% CI 24–94) vs. 30 months (95% CI 0–70) for the FOLFOX group. The Kaplan–Meier method did not show any statistically significant difference between the two groups in overall survival (log-rank test. *P* = 0.705; Figure 5).

### 3.10. Metastasectomy

Nine patients underwent metastasectomy, including three liver mets, two lung mets, three peritoneal/retroperitoneal pelvic mets, and one ovarian mets. The median OS for these patients was 70 months (95% CI 59.6–82).

### 3.11. MSI-H vs. MSI Stable

Seven patients had MSI-high tumours, while 24% (27) had MSI-stable. The 2-year OS was 83% and 75% for MSI-H and MSS, respectively. Around 70% (81 patients) had unknown MSI status. Two patients received pembrolizumab as a second-line treatment, and one of them had ongoing treatment for more than 2 years. 

### 3.12. Prognostic Factors

Multivariate Cox regression analysis showed only poor performance status at diagnosis as a predictor for shorter overall survival (*p* < 0.05).

Univariate analysis didn’t show any significant correlation between RFS and perineural invasion, lymphovascular invasion, differentiation, site of the tumour, or bowel obstruction. There was a significant association between death and presentation with bowel obstruction (*p* = 0.043).

## 4. Discussion

Clinical research on the management and outcomes of SBA is limited due to the paucity of cases. The present study explores the outcomes and clinicopathological features of 115 SBA cases seen at the Ottawa Hospital over the past 10 years. Although a good number of case series were published, data from Canada about SBA is limited, with relatively small study populations. The incidence of SBA has increased steadily over the past decades, and it is now estimated that the incidence is 3.3 per 100,000 in Canada. This is in keeping with an increasing trend in SBA incidence over the last decade reported from the SEER database [19,20,21].

The characteristics of the study’s cohort are similar to those reported from the Canadian series, such as age, gender, and ECOG status. However, there were notable differences in the TNM stage at presentation and the proportion of duodenal and jejunal tumours. For instance, the percentage of stage IV at diagnosis in our study was 58%, in contrast to 31% from a cohort of the Princess Margaret Hospital (PMH). Moreover, the percentage of duodenal tumours in our study is 61%, while the cohort from British Columbia showed that 38% of patients had the disease in the duodenum [19,22].

The majority of patients with localised SBA undergo curative surgery. Our centre has a comparable resectability rate to that of other centres. In our study, the median OS was 94.4 months vs. 30.1 months for the curative surgery group compared to the no-surgery group. This confirms the pivotal role of surgical resection in the management of SBA.

The role of adjuvant therapy for SBA was assessed only through data from retrospective reports or meta-analyses. These studies have shown mixed efficacy of adjuvant therapy (either chemotherapy or chemoradiotherapy), with some showing a benefit to adjuvant therapy. BALLAD trial is an ongoing phase III study investigating the role of adjuvant 5-FU/leucovorin (5-FU/LV) or 5- FU/LV plus oxaliplatin (FOLFOX) compared to observation alone for patients with stage I-III SBA. The results of this study should shed light on the adjuvant therapy approach in the coming years [23,24,25]. Patients with stage II MSI- H/dMMR SBA may have a good prognosis, yet the benefit of adjuvant therapy is unclear. In our study, only 17 patients received adjuvant chemotherapy, with eight subsequently having a recurrence of their disease.

Numerous retrospective studies have indicated that palliative chemotherapy for SBA offers survival benefits compared with palliative care alone. Median OS was between 11 and 15 months for patients treated with chemotherapy and between 4 and 7 months for patients receiving only supportive care [19,26,27]. In our study, 57 patients (49.5%) received palliative systemic therapy, while 18 (16%) had supportive care only. The median OS was 34 (95% CI 0–74) months for all patients with stage IV. However, this result overestimates OS due to a lack of documentation of death in some patients’ charts. There is no randomised trial comparing the efficacy of different chemotherapy regimens in advanced SBA. FOLFOX and CAPOX were evaluated as first-line palliative therapies in two phase II studies, and response rates of around 50% were reported, with a median PFS of 7.8 and 11.3 months and median OS of 15.2 and 20.4 months [28,29]. 

The AGEO study assessed FOLFIRI regimens as second-line therapy. A tumour control rate of around 52% was reported with PFS at 3.2 months [30]. MMR/MSI status may guide the choice of second-line therapy in SBA. Pembrolizumab or nivolumab, with or without ipilimumab, as second-line treatment options for dMMR/MSI-H, advanced SBA are recommended by NCCN guidelines based on positive results for CRC and SBA [18], [31,32,33]. In our study, 57 patients (49.5%) received palliative systemic therapy, while 16% (18) received supportive care only. The median OS was 34 (95% CI 0–74) months for stage IV. However, this result overestimates OS due to a lack of documentation of death in patients’ charts. 

The combination of FOLFIRI followed by FOLFOX6 or the reverse sequence is a well-established treatment approach for advanced colorectal cancer. Both sequences achieved similar efficacy and prolonged survival [34]. However, there is no available data about the efficacy of this approach in SBA. In our study, 20% (23) received FOLFOX chemotherapy as the initial treatment, while 17% (19) received FOLFIRI. The median OS for the FOLFIRI group was 59 months (95% CI 24–94) vs. 30 months (95% CI 0–70) for the FOLFOX group. Kaplan–Meier method didn’t show any statistically significant difference between the two groups in overall survival (log-rank test, *p* = 0.705). This might support the similarity of this approach to CRC management, yet more prospective data are needed.

The benefit of surgical treatment for resectable metastatic SBA has not been evaluated in prospective clinical studies. Role of Cytoreductive surgery (CRS) plus hyperthermic intraperitoneal chemotherapy (HIPEC) for patients with peritoneal metastases (PM) from SBA evaluated in a retrospective study and showed prolonged survival for selected patients; with survival rates of 83.2% at 1 year, 46.4% at 3 years, and 30.8% at 5 years [35]. In our study, nine patients underwent mastectomy for solitary metastases. The Median survival for these patients was 70 months (95% CI 59.6–82). This is a relatively higher median OS than that for advanced SBA managed with chemotherapy alone.

A high number of second malignancies (SM) has been observed in patients with SBA. Weinerman and Ripley reviewed the tumour registries of two Canadian provinces (British Columbia and Manitoba) for SBA. They noted a more than eightfold increase in SM with SBA. They hypothesised that the lack of repair or unstable genes could be the cause of these conditions. In our study, 28 patients (24%) had a diagnosis of additional primary cancer prior to or after SBA diagnosis, and colon cancer (36%) was the most common SM. More research is needed to investigate whether there is an association between SBA and other cancers.

Our study has several limitations. These include a retrospective design, lack of pathology records and biomarkers data for some patients, and the fact that 33 patients likely died, but we could not find documentation of death in medical records. This certainly has affected our analysis, especially for the estimation of OS. The findings of OS in this study should be interpreted with caution. Despite these limitations, we believe our study provides valuable insight into SBA and its outcome.

## 5. Conclusions

Small bowel adenocarcinoma (SBA) remains one of the most aggressive tumours with a dismal prognosis even after surgical resection. The optimal chemotherapy regimen has not been established. Further studies are needed to explore the role of adjuvant chemotherapy for stages I-III SBA.

## Figures and Tables

**Table 1:** Baseline patient’s characteristics.

	Total	*n* = 115 (Percent %)	
**Age**	Mean Median	65 67	
**Gender**	Male	67	58
	Female	48	42
**Comorbidities**	HTN	50	44%
	DM	22	19%
	DLP	30	26%
	IBD	6	5%
	Lynch / MSI-Hi		
	Other	60	52%
	2nd cancer	28	24%
**Clinical presentation**	Incidental/screening	4	4%
	Abdominal pain	55	48%
	Nausea/vomiting	41	36%
	GI bleeding	14	12%
	Anaemia	29	25%
	Bowel obstruction	27	24%
	Gastric outlet obstruction	16	14%
	Perforation	3	3%
	Weight loss	36	31%
	Other	86	75%
**Family history of GI cancer**		25	22%
**Familial/FAP**		6	5%
**Duration of symptom**	<14 days	16	14%
	>14 days	15	13%
	>2 months	28	24%
	>6 months	33	29%
	Screening/incidental	4	4%
	NA	19	17%
**ECOG**	0	15	15%
	1	58	50%
	2	18	16%
	3	15	30%
	NA	9	8%
**Mode of initial diagnosis**	CT	52	45%
	Endoscopy	26	23%
	Surgical exploration	4	4%
	Other	7	7%
	NA	26	27%
Curative Surgical resection		48	42%
Observation/supportive care only		18	16%

Abbreviations: HTN: Hypertension, DM: Diabetes mellitus, DLP: Dyslipidemia, IBD: inflammatory bowel disease, MSI-HI: microsatellite instability-high, GI: Gastrointestinal, FAP: Familial adenomatous polyposis, ECOG: Eastern Cooperative Oncology Group performance status, NA: Not available, CT: computerised tomography.

**Table 2:** Pathology and staging data.

Pathology			
	Total	*n* = 115 (Percent %)	
Site	Duodenum	70	61%
	Jejunum	19	17%
	Ileum	11	10%
	Small bowel NOS	15	13%
Differentiation	well	12	10%
	Moderate	33	29%
	Poor	17	15%
	Unknown	53	46%
Signet ring		8	7%
Mucinous		8	7%
RAS/RAF/MSI	RAS mutant Wild unknown	10 11 94	9% 10% 81%
	BRAF mutant Wild unknown	3 13 99	3% 11% 86%
	MSI stable MSI-high unknown	27 7 81	24% 6% 70%
TNM stage	I	8	7%
	II	16	14%
	III	25	22%
	IV	64	58%
	NA	2	2%
Sites of mets **	Liver	30	
	Peritoneum	36	
	Lung	18	
	Lymph nodes	14	

** stage IV *n* = 64 in addition to 16 patients who had metachronous metastasis. Abbreviations: NOS: Not otherwise specified. MSI: microsatellite instability, NA: Not available.

## Data Availability

The data that support the findings of this study are available from the corresponding author upon reasonable request.
